# Presentation and Management of Silicone Lymphadenopathy: A Single Institutional Retrospective Cohort Study

**DOI:** 10.1007/s00266-024-04649-z

**Published:** 2025-01-10

**Authors:** Anshumi Desai, Taylor Smartz, Orel Tabibi, Peter A. Borowsky, Kashyap Komarraju Tadisina, Devinder P. Singh, Susan B. Kesmodel, Kristin E. Rojas, Juan R. Mella-Catinchi

**Affiliations:** 1 Division of Plastic and Reconstructive Surgery, DeWitt Daughtry Family Department of Surgery, Miami, FL USA; 2 University of Miami Miller School of Medicine, Miami, FL USA; 3 Division of Surgical Oncology, DeWitt Daughtry Family Department of Surgery, Miami, FL USA; 4 Sylvester Comprehensive Cancer Center, Miami, FL USA

**Keywords:** Silicone lymphadenopathy, Breast cancer, Breast augmentation, Breast reconstruction, Siliconoma

## Abstract

**Introduction:**

Silicone Lymphadenopathy (SL) is a complication of breast implants that involves migration of silicone to nearby soft tissue/lymph nodes. Data on its clinical features and management is scarce. We aimed to identify the clinical presentation and management of SL.

**Methods:**

A single-institution retrospective cohort study was conducted from our institutional imaging system where search terms “Silicone lymphadenopathy”, “silicone adenitis” and “silicone adenopathy” were used to identify patients with SL (January 2016–September 2023). Patient demographics, clinical features, imaging findings, pathological investigation, and treatment were obtained from the medical records.

**Results:**

Of 52 patients with SL, breast augmentation accounted for 90.4% of the implant placements. All patients had silicone implants placed at some time. A significant portion of patients (69.3%) were asymptomatic, while 7.7% had non-tender lymphadenopathy, 19.2% experienced painful lymphadenopathy, and 1.9% presented with mixed symptoms. Implant rupture was observed in 88.7% of cases; 13.0% intracapsular, 26.1% extracapsular, 15.2% both, and unknown in 45.7%. Axillary nodes were the most commonly involved (86.5%), and ultrasonography was most commonly used to detect SL (80.7%). Biopsy was performed in 17.3% of cases, confirming benign pathology in all cases. No patients required surgical excision of lymph nodes for management of SL.

**Conclusion:**

Most patients with SL are asymptomatic and are managed with observation. Biopsy and surgical intervention should be reserved for those patients with abnormal imaging or persistent symptoms. Evaluation of lymphadenopathy is essential to exclude malignancy in patients with a history of breast cancer.

**Level of Evidence III:**

This journal requires that authors assign a level of evidence to each article. For a full description of these Evidence-Based Medicine ratings, please refer to the Table of Contents or the online Instructions to Authors www.springer.com/00266.

## Introduction

Breast augmentation is the most frequently performed cosmetic plastic surgery procedure as reported by the American Society of Plastic Surgeons (ASPS) [1]. In 2022, ASPS Member Surgeons performed 298,568 breast augmentations [1]. In addition, about 80% of post-mastectomy breast reconstruction procedures are implant-based [2].

Since their introduction in the 1960s, silicone breast implants have become a mainstay for both breast augmentation and reconstructive surgeries [3]. Innovations in implant technology and design have continuously improved their safety profile and effectiveness, leading to a surge in their utilization [4]. Despite this, patients with breast implants require routine long-term care to screen for breast implant-related symptoms and pathology [5]. Silicone lymphadenopathy (SL) remains an infrequent yet acknowledged complication associated with silicone breast implants. This condition typically arises when silicone from damaged implants migrates into adjacent tissues, often via macrophages, and accumulates in the lymph nodes [6, 7]. It elicits a persistent foreign body reaction and results in lymph node enlargement. The clinical presentation of SL can be variable, with its incidental discovery sometimes occurring during routine screening or as part of the assessment of symptoms reported by patients.

Despite a growing number of breast implant procedures, there is a notable gap in the literature on the incidence of SL and strategies for its management. Current literature is mainly composed of isolated case reports and small case series. No clear consensus exists on the management or the need for surgical intervention for SL. Our study, based in Miami, Florida, a region with a high volume of breast surgeries, frequently encounters complications in patients coming from Latin America [8]. The authors have previously conducted a systematic review of SL which showed that it should prompt thorough evaluation to rule out malignancy, and personalized management discussions should be conducted to optimize patient care. To help reduce the existing knowledge gap, this institutional study serves to provide additional granular information on the clinical presentation and management of patients with SL [9].

## Materials and Methods

### Patient Selection

A single-institution retrospective cohort study was conducted from January 2016 to September 2023 after Institutional Review Board (IRB) approval was granted. Three search terms “Silicone lymphadenopathy”, “silicone adenitis” and “silicone adenopathy” were used to identify patients with SL from our institutional imaging system.

### Clinical Radiologic and Pathologic Findings

Patient demographics, clinical presentation, imaging findings, pathological investigation, and treatment were obtained from the electronic medical records. Clinical notes were reviewed to identify details of patient symptoms and presentation. Radiology reports including mammograms, ultrasound, magnetic resonance imaging (MRI), and computed tomography (CT) Scans were reviewed to identify imaging features including evidence of implant rupture and the presence of SL. Pathology reports of patients who underwent biopsy were reviewed to identify the presence of malignancy or benign disease.

### Statistical Analysis

Descriptive statistics were used to analyze the data. Frequency and percentages were used to summarise all findings.

## Results

**Fig. 1:**
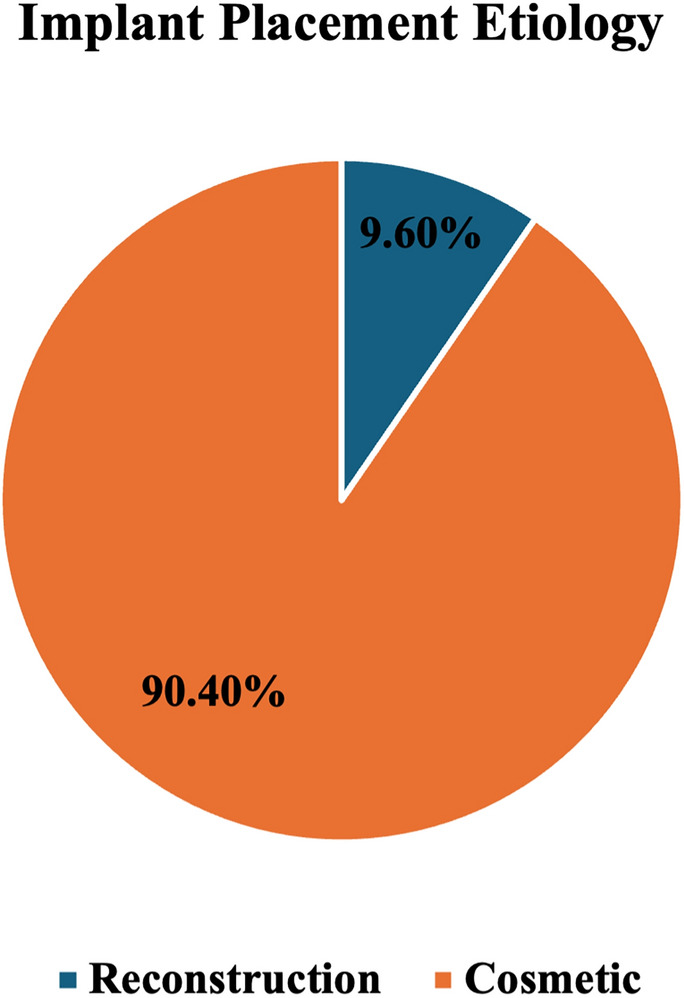
Distribution of implant placement etiology

52 patients with SL were identified. The median age was 57 years [SD = 12.17] and the median body mass index (BMI) was 24.72 kg/m^2^ [SD = 4.70]. Patient demographics are described in Table 1. Most patients were white 94.2% (*n* = 49) and 73.1% (*n* = 38) identified as Hispanic. Only 15.4% (*n* = 8) of patients had a history of breast cancer and 3.8% (*n* = 2) had a breast cancer recurrence diagnosed at the time of detection of SL. Implants were placed for cosmetic purposes in 90.4% (*n* = 47) of the cases, while 9.6% (*n* = 5) had implants for reconstruction (Fig. 1). The time from initial implant placement to SL was known in 83% (*n* = 44) of patients, and the median time to SL detection was 12.5 years [SD 11.15].

**Table 1:** Patient clinical and sociodemographic characteristics

Age (years) Median (SD)	57 (SD 12.17)
BMI kg/m^2^ Median (SD)	24.72 (SD 4.70)
Race *n* (%)	
White	49 (94.2%)
Black	0 (0.0%)
Other	3 (5.8%)
Ethnicity *n* (%)	
Hispanic	38 (73.1%)
Non-Hispanic	13 (25.0%)
Unknown	1 (1.9%)
Prior history of breast cancer *n* (%)	
Yes	8 (15.4%)
No	44 (84.6%)
Current breast cancer *n* (%)	
Yes	2 (3.8%)
No	50 (96.2%)
Reason for implant placement *n* (%)	
Reconstruction	5 (9.6%)
Cosmetic	47 (90.4%)

BMI= body mass index

**Fig. 2:**
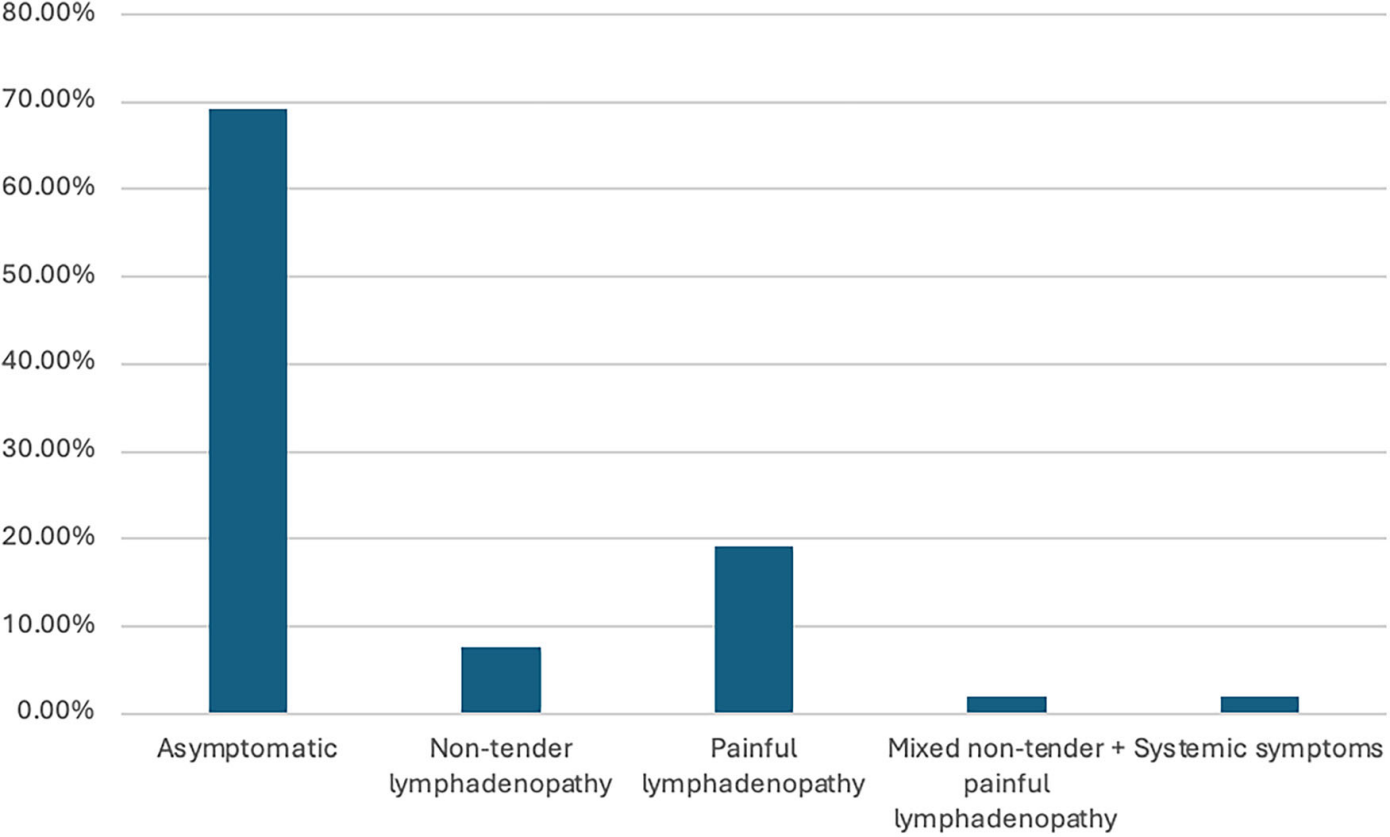
Clinical presentation of patients detected with SL

**Fig. 3:**
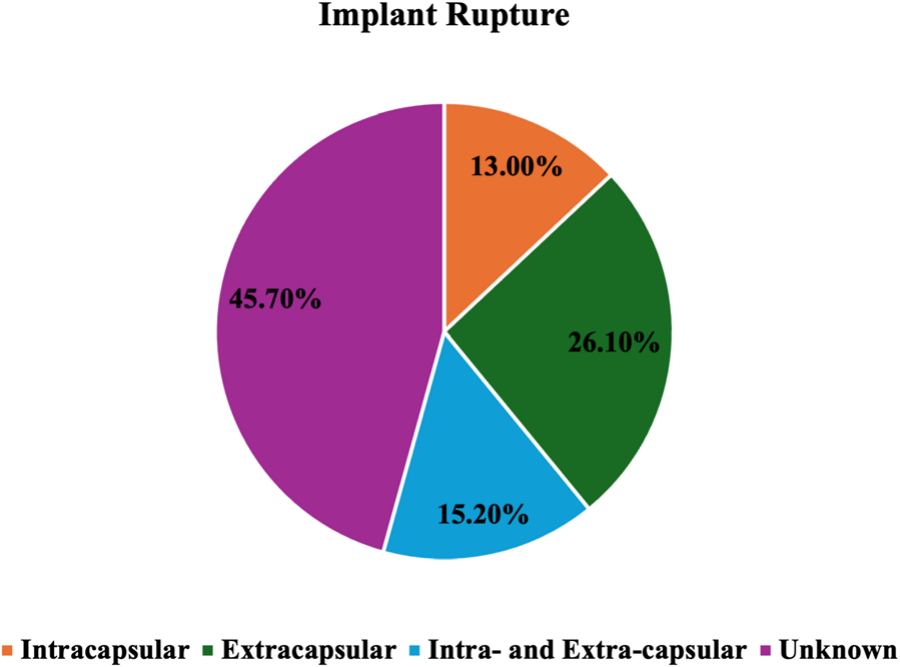
Type of implant rupture in patients with SL

Clinical presentation and imaging characteristics for patients with SL are presented in Table 2. While most patients presented with unilateral SL (71.1%, *n* = 37), 28.9% (*n* = 15) of patients had bilateral SL. Most patients, 96.2% (*n* = 50), had silicone implants in place at the time of SL diagnosis, while 3.8% (*n* = 2) had saline implants and had a history of prior silicone implants. Most patients (69.3%, *n* = 36) were asymptomatic (Fig. 2), while non-tender lymphadenopathy was reported in 7.7% (*n* = 4), painful lymphadenopathy in 19.2% (*n* = 10), and mixed non-tender and painful lymphadenopathy in 1.9% (*n* = 1). None of the patients had capsular contracture, breast asymmetry, respiratory symptoms, or pericarditis. Systemic symptoms including fever, chills, weight loss, generalized weakness, and fatigue were only noted in one patient. Implant rupture was evident in 88.7% (*n* = 46) of patients; 13.0% (*n* = 6) intracapsular, 26.1% (*n* = 12) extracapsular, and 15.2% (*n* = 7) both intra- and extra-capsular. The type of rupture was unknown in 45.7% (*n* = 21) of cases (Fig. 3). Of patients with documented implant rupture, 34.6% (*n* = 18) had a rupture of the current implant and 53.9% (*n* = 28) had a rupture of a prior implant which was subsequently removed/exchanged. Of the 11.5% (*n* = 6) patients that did not have an implant rupture, the capsules were noted to be intact in all 6. Lymph node region involvement was predominantly in the axilla at 86.5% (*n* = 45), with 3.9% (*n* = 2) in the internal mammary and 9.6% (*n* = 5) involving more than one region. SL was detected by ultrasonography (USG) in 80.7% (*n* = 42), mammography (MMG) in 67.3% (*n* = 35), magnetic resonance imaging (MRI) in 17.3% (*n* = 9), and computed tomography (CT) in 3.8% (*n* = 2) of cases. Multiple imaging modalities were employed in 42.3% (*n* = 22) of cases.

**Table 2:** Clinical presentation and imaging for patients with SL

	*n* (%)
Laterality of SL	
Right	18 (34.6%)
Left	19 (36.5%)
Bilateral	15 (28.9%)
Implant type at the time of SL detection	
Silicone	50 (96.2%)
Saline^+^	2 (3.8%)
Symptoms	
Asymptomatic	36 (69.3%)
Non-tender lymphadenopathy	4 (7.7%)
Tender lymphadenopathy	10 (19.2%)
Mixed non-tender + tender lymphadenopathy	1 (1.9%)
Systemic symptoms	1 (1.9%)
Implant rupture	
Yes	46 (88.7%)
No	6 (11.3%)
Type implant rupture	
Intracapsular	6 (13.0%)
Extracapsular	12 (26.1%)
Intra- and Extra-capsular	7 (15.2%)
Unknown	21 (45.7%)
Timing of implant rupture	
Current	18 (34.6%)
Prior	28 (53.9%)
No rupture	6 (11.5%)
LN region involved with SL	
Axilla	45 (86.5%)
Internal mammary	2 (3.9%)
More than one	5 (9.6%)
Imaging to detect SL^++^	
USG	42 (80.7%)
MMG	35 (67.3%)
MRI	9 (17.3%)
CT	2 (3.8%)
More than one	22 (42.3%)

*SL* silicone lymphadenopathy, *USG* ultrasonography, *MMG* mammography, *MRI* magnetic resonance imaging; CT, computed tomography; PET,positron emission tomography;

+ These two patients had a history of prior silicone implant placement

++ percentage will not sum up to 100% as patients had more than one imaging modality

**Fig. 4:**
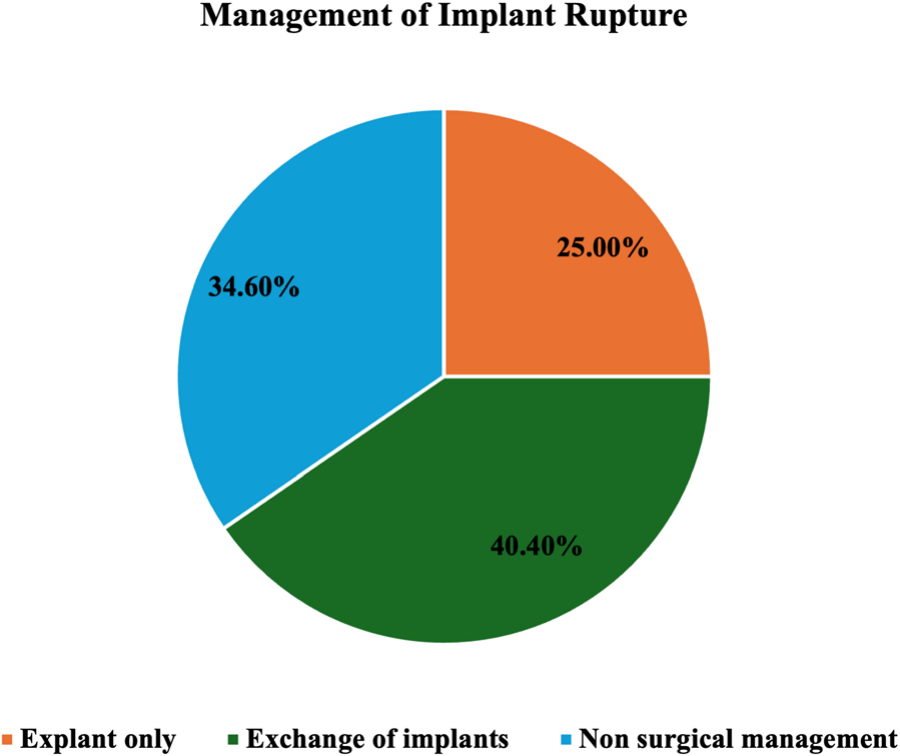
Management of implants

The management of SL among the study patients is described in Table 3. A biopsy was performed in 15.4% (*n* = 8) of cases due to suspicious imaging features. This included fine needle aspiration cytology (FNAC) in 12.5% (n = 1), and core biopsy in 87.5% (*n* = 7) of patients. Histopathologic examination confirmed the diagnosis of SL with benign features in 87.5% (*n* = 7) of those biopsied, whereas 12.5% (*n* = 1) patients did not have histopathology suggestive of SL. None of the patients who underwent biopsy had histopathological features of malignancy. Management of current or prior implant rupture was explantation in 25.0% (*n* = 13), implant exchange in 40.4% (*n* = 21), and non-surgical management in 34.6% (*n* = 18) of cases. These included surgeries done before as well as at the time of SL detection (Fig. 4). Of the 18 patients with nonsurgical management, intact implants were noted in 6 patients. In the remaining 12 patients, 10 patients did not follow up, one patient had an intact capsule on subsequent imaging, while 1 was followed up with extracapsular silicone without implant exchange or removal.

**Table 3:** Management of patients with SL

Biopsy	*n* (%)
Yes	8 (15.4%)
No	44 (84.6%)
Type of biopsy	
FNAC	1 (12.5%)
Core biopsy	7 (87.5%)
Implant management	
Explant	13 (25.0%)
Implant exchange	21 (40.4%)
Non-surgical management	18 (34.6%)

FNAC= fine needle aspiration cytology

## Discussion

SL is an infrequent phenomenon following implant-based breast surgeries. Frequently identified incidentally, the actual prevalence of SL likely surpasses the reported numbers [10]. There have been a few case reports and case series that have addressed SL. To our knowledge, this is the largest study that reports clinical presentation, imaging findings, and management of SL.

Of the 52 patients with SL in our study, 90.4% underwent implant procedures for breast augmentation, while the remaining 9.6% received implants for reconstructive reasons. The discovery of lymphadenopathy in patients with a prior diagnosis of breast cancer necessitates a thorough assessment for cancer recurrence [11]. In patients who had implant placement for breast augmentation, while malignancy should also be excluded, the presence of SL should be considered. Moreover, patients undergoing reconstructive breast surgery often have a lack of breast tissue and may not receive screening mammograms/MRIs as regularly as those who have undergone breast augmentation procedures. This could be why we saw a distinctly higher incidence of cases in cosmetic procedures compared to reconstructive procedures [9]. The median time of detection was 13.5 years for reconstructive procedures and 12.5 for cosmetic procedures which was not significantly different.

Silicone implants have recently become the preferred choice due to the cohesiveness reflecting a trend observed in the industry [12]. As expected, there is a higher incidence of SL with silicone implants [13]. They represent the majority of implants used in both aesthetic and reconstructive surgeries [14]. In the present study, all patients had current or prior silicone implants resulting in SL which is consistent with prior studies. Despite this, it should be recognized that saline implants carry a risk of SL due to their silicone-containing shells which may leak into adjacent tissue, causing the spread of silicone [15]. Therefore, healthcare providers should consider SL as a potential diagnosis when a patient with any type of implant reports lymph node swelling. With either silicone or saline implants, the incidence of implant failure or rupture can be up to 16% within a decade of implant placement. Implant failure has been associated with SL [16]. Most patients in our study also had a current or prior implant rupture. However, SL may be present even in patients with intact breast implants due to silicone leakage from the implant shell [17]. Intact implants were present in 11.3% cases of in our cohort. The rupture can be intracapsular, extracapsular, or a combination of both. However, the type of rupture does not indicate the presence of SL.

SL was detected most commonly in asymptomatic patients in our study (69.3%), consistent with prior studies [9]. Of the symptomatic patients, most presented with tender lymphadenopathy, 19.2% of cases. According to the authors’ prior systematic review, the clinical manifestations of SL can be quite variable, ranging from asymptomatic to systemic symptoms like fever, generalized discomfort, unintended weight reduction, and muscle aches. In addition, it may be difficult to differentiate whether the symptoms are due to SL or breast implant illness [18]. The most commonly observed symptom is lymph node swelling with or without tenderness, although other symptoms such as discomfort, changes in breast shape, and pronounced systemic symptoms have also been observed [19]. These cases highlight the potential of SL to mimic the signs of cancer, making it critical to conduct a comprehensive evaluation of patients with these symptoms. Providers should also balance the likelihood of SL against other potential diagnoses to prevent unwarranted medical testing and the associated costs, particularly in those with a lower risk [19]. The most common location of SL in our study was the axilla in 86.5% of cases. This is similar to the findings from the authors’ previous systematic review which showed that axillary lymphadenopathy was the most affected region (72%), followed by internal mammary (21%), cervical/supraclavicular (19%), and mediastinal (13%) regions [9].

SL is rarely felt on clinical examination and is most commonly detected on imaging that is done for breast cancer screening or surveillance. Patients with breast implants for augmentation get follow-up ultrasound or MRI usually 5-6 years after implant placement or earlier if they are symptomatic. In 2006, the United States Food and Drug Administration (FDA) advised periodic screening for patients with silicone-filled implants to detect possible rupture. The FDA suggests utilizing breast MRI for asymptomatic patients to evaluate implants 5–6 years after implantation and then every 2–3 years thereafter [5]. However, MRI is not always available and is very expensive. Ultrasound is an easy-to-use, readily available, and relatively inexpensive imaging modality that can be used as an alternative to breast MRI to detect implant-related complications [20]. Mammograms, MRIs, and CTs can provide additional information for the confirmation and characterization of lymphadenopathy [21]. In our patients, SL was detected by ultrasound 80.7% of the time. A “snowstorm” appearance of lymph nodes on ultrasound is diagnostic of SL [22]. The sensitivity and specificity of this ultrasound-based diagnosis, utilizing the snowstorm sign, are reported at 87.5% and 100%, respectively [21]. Consequently, in individuals with low-risk factors and exhibiting painless lymphadenopathy, the identification of a snowstorm appearance on ultrasound may be sufficient for diagnosis and would reduce the necessity for additional interventions like biopsy [21, 22]. Telegrafo et al. report the sensitivity and specificity of ultrasound as 50–77 and 55–84%, respectively, in detecting implant rupture. It can detect intracapsular as well as extra-capsular rupture [23]. Their study found that ultrasound had 63% sensitivity, specificity, and accuracy for intracapsular rupture and 100% sensitivity for extracapsular rupture. Hence, ultrasound is effective as an initial screening, with MRI needed for intra-capsular rupture [23]. According to the economic analysis conducted by Chung et al., ultrasound screening followed by MRI was found to be the most effective approach for asymptomatic women, while ultrasound alone was considered optimal for symptomatic women [24]. Patients with implants also get a mammogram as part of their cancer screening protocol after the age of 40 [25]. 67.3% of our patients had SL detected on mammograms. This might be the first modality to detect enlarged lymph nodes that could be SL which can be further evaluated by ultrasound. Once SL is detected on imaging, biopsy may be performed for definitive diagnosis particularly if there are concerning imaging features or a history of breast cancer [26]. The histological appearance of silicone is marked by refractile, unstained droplets under light microscopy, and in SL cases, where silicone is within lymph nodes, there is chronic inflammation from a foreign body response [7, 27]. Biopsy can be performed by either fine needle aspiration or core biopsy. Most of the patients with SL in our study who had biopsy, underwent core biopsy. Core biopsy provides more tissue for analysis and is more sensitive in the detection of cancer. Excisional biopsies are usually only performed when patients are already scheduled for implant removal and exchange due to implant rupture. None of the patients in our study had an excision for the SL alone. In instances of newly detected lymphadenopathy, the potential for a new cancer diagnosis or cancer recurrence should be among the differential diagnosis. This is especially pertinent for individuals with a history of prior cancer, as recurrence remains a distinct possibility [11]. Therefore, when new lymphadenopathy is detected in the presence of a new cancer diagnosis or history of cancer, a lymph node biopsy is necessary to provide a definitive diagnosis [11].

None of the patients in the present study had surgical excision alone of the SL for management. Implants with rupture were managed most commonly by either implant removal or implant exchange. The most common indication for implant exchange was implant rupture, which aligns with the current literature [28]. Prior studies have shown mixed results from excision of SL with some showing complete resolution of symptoms while others have shown recurrence or persistent symptoms [29–31]. Therefore, it is crucial to emphasize that the excision of affected nodes has produced varied outcomes in terms of addressing patient symptomatology. If patients present with painful lymphadenopathy surgical excision should be considered. However, excision is generally not recommended in asymptomatic patients due to the potential risk of lymphedema [32]. It is also crucial to recognize that removing implants does not cure SL, nor does it prevent the development of future lymphadenopathy [30]. Moreover, during the removal of breast implants, there is a risk of spreading silicone, which could exacerbate the patient's symptoms [33]. Therefore, it is essential to have a thorough discussion with patients about these risks and potential outcomes as part of the decision-making process. Treatment plans should be customized based on the imaging results and the patient's clinical history. Accordingly, individuals with SL who do not have a history of breast cancer are not required to undergo additional monitoring beyond standard breast cancer screening guidelines.

This is a retrospective study conducted at a single institution. The screening protocols implemented at our tertiary care academic center may not be generalizable to smaller hospitals. Given that this condition is not commonly encountered, the number of cases will inherently remain limited. The search terminology used might have missed a few cases that were reported as reactive lymphadenopathy. A multi-institutional study would allow for a larger and more diverse patient population, thereby enhancing the generalizability of the findings.

## Conclusion

SL is a complication associated with breast implants. Most patients are asymptomatic and SL is detected on screening. Therefore, a conservative approach is recommended for SL for most patients. Biopsy and surgical intervention should be reserved for those with abnormal imaging, persistent symptoms, or evidence of implant rupture. In patients with a history of breast cancer, evaluation of lymphadenopathy is essential to exclude malignancy.
